# Checklist of host plants of insect galls in the state of Goiás in the Midwest Region of Brazil

**DOI:** 10.3897/BDJ.3.e6835

**Published:** 2015-11-13

**Authors:** Walter Santos de Araújo, Eder Dasdoriano Porfírio Júnior, Bárbara Araújo Ribeiro, Taiza Moura Silva, Elienai Cândida e Silva, Frederico Augusto Guimarães Guilherme, Claudia Scareli-Santos, Benedito Baptista dos Santos

**Affiliations:** ‡ Universidade Federal de Goiás, Goiânia, Brazil; § Ministério do Meio Ambiente, Brasília, Brazil; | Universidade Federal de Goiás, Jataí, Brazil; ¶ Universidade Federal do Tocantins, Araguaína, Brazil

**Keywords:** Cerrado, galls, Goiás, host plants, Neotropical savannas, plant-insect interactions

## Abstract

**Background:**

Surveys of host plants of insect galls have been performed in different regions of Brazil. The knowledge of species of host plants of insect galls is fundamental to further studies of plant-galling insect interactions. However, a list of host plant species of gall-inducing insects has not yet been compiled for the flora of the Midwest Region of Brazil.

**New information:**

We provide a compilation of the plant species reported to host insect galls in the Cerrado of the state of Goiás in the Midwest Region of Brazil. Altogether we found records for 181 species of 47 families of host plants, which hosted 365 distinct gall morphotypes.

## Introduction

Insect galls are structures formed by the development of larvae or nymphs in the interior of plant tissues (Mani 1964, Rohfritsch 1982, Stone and Schönrogge 2003). Galls develop by hypertrophy and hyperplasia of the plant cells, which changes all of the structure of the attacked plant organ (Moura et al. 2008). Because of the intimate level of interaction between gall-inducing insects and their host plants at histological and cellular scale, these insects are considered the most specialized guild of herbivores (Stone and Schönrogge 2003, Carneiro et al. 2009, Fernandes and Santos 2014). Due to this high degree of specialization, knowledge regarding the identity of host plant species is fundamental to studies of plant-galling insect interactions.

It is already known that there exists a high diversity of insect galls in the Neotropics (Gagné 1994, Fernandes and Santos 2014), mainly in the savannas located in central Brazil (Araújo et al. 2014b). A likely explanation for this is the great diversity of plants in the region (Espírito-Santo and Fernandes 2007), since richness of host plants is an important factor in explaining the distribution patterns of insect galls (Araújo et al. 2014b). Considering that each species of plant is a potential host for galling, a greater local and regional diversity of plant species implies greater galling species richness (Mendonça 2007). Several studies in Brazil have produced local and regional lists of host plant species and their associated galls, especially in the South and Southeast regions (*e.g.* Gonçalves-Alvim and Fernandes 2001, Maia and Fernandes 2004, Mendonça 2007, Maia et al. 2008, Bregonci et al. 2010), while in other regions, such as the Midwest, surveys are still scarce (Araújo et al. 2013). In the present work, we provide a compilation of plant species that have been recorded to host insect galls in the state of Goiás in the Midwest Region of Brazil.

## Material and methods

**Figure 1.**
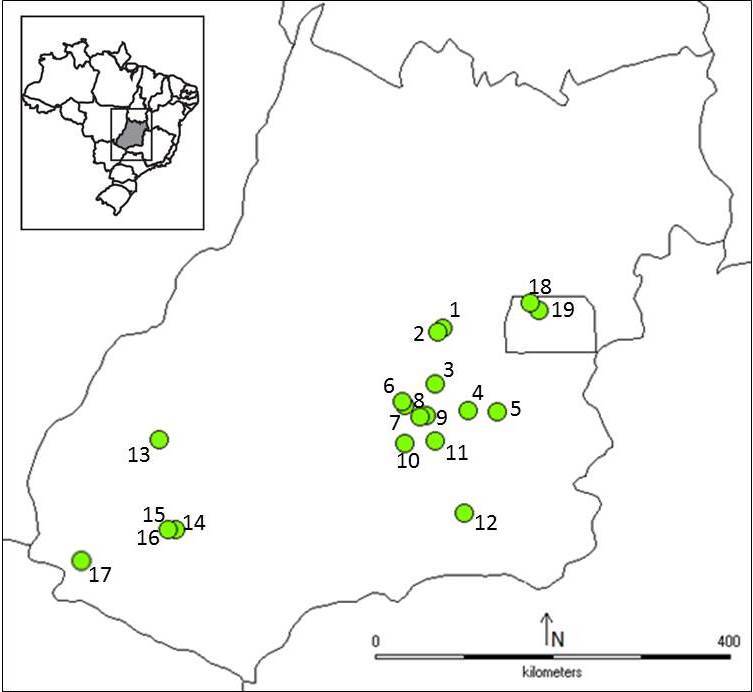
Localization of the sites in which insect galls were sampled in the state of Goiás, Midwest, Brazil. 2005-2013. The number of each site corresponds to code in the Table 1.

**Figure 2.**
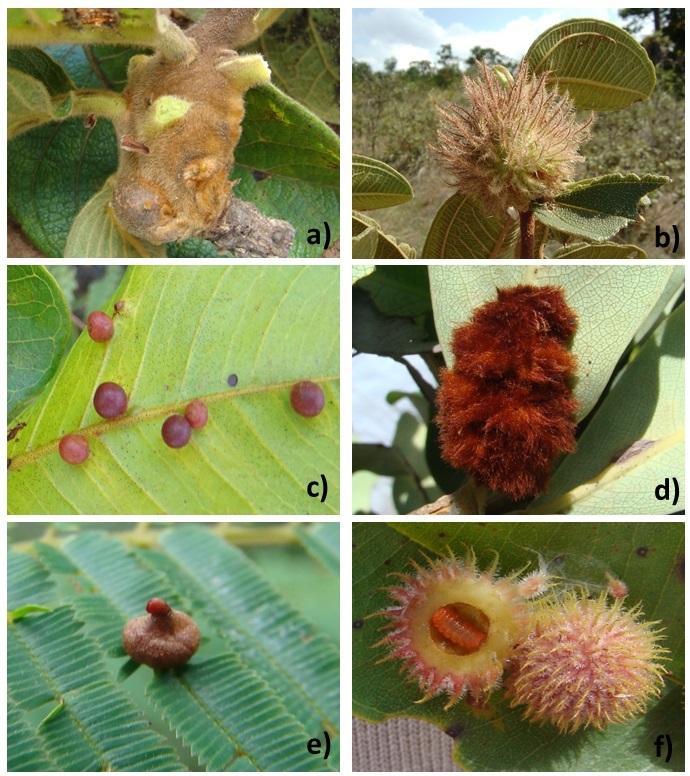
Examples of insect galls recorded to state of Goiás, Midwest, Brazil. 2005-2013. a) Stem gall of *Apion* sp. (Coleoptera, Brentidae) on *Diospyros hispida* (Ebenaceae); b) Stem gall of *Palaeomystella oligophaga* (Lepidoptera, Coleophoridae) on *Macairea radula* (Melastomataceae); c) Leaf galls of *Neotrioza* sp. (Hemiptera, Psyllidae) on *Psidium salutare* (Myrtaceae); d) Leaf gall of *Myrciariamyia admirabilis* (Diptera, Cecidomyiidae) on *Erythroxylum suberosum* (Erythroxylaceae); e) Leaf gall of an undetermined Cecidomyiidae (Diptera) on *Anadenanthera peregrina* (Fabaceae); f) Leaf galls of an undetermined Cecidomyiidae (Diptera) on *Qualea parviflora* (Vochysiaceae) with the detail of a larva in the internal chamber.

We compiled data from different surveys of plant species that are hosts of insect galls from several locations in the state of Goiás in the Midwest Region of Brazil (Fig. 1). We also included data from the Distrito Federal, which is a separate political entity, but is surrounded by the state of Goiás. All records of hosts and galls were collected between 2005 and 2013 in different types of vegetation that comprise the Cerrado biome (Table 1). We included data published in local checklists (*e.g.* Santos et al. 2010, Araújo et al. 2011, Araújo et al. 2014a) as well as unpublished data. The identification of the plants was made by comparison with the collections of Universidade Federal de Goiás, herbarium, literature, as well as consultation with specialists. We checked the synonymy using The Plant List database (www.theplantlist.org). In addition to the list of host plants, we provide a short morphological characterization of gall morphotypes (plant organ, gall form and gall color) associated with each host plant species. The use of gall morphotypes is a commonly used and reliable parameter because evidence indicates that each gall morphospecies (examples in Fig. 2) is unique to a particular gall-inducing insect (Stone and Schönrogge 2003), and each galling species is specific to a particular host plant (Abrahamson et al. 1998). Insect galls can be differentiated from galls induced by other organisms, such as mites and nematodes, because they form an internal chamber where the immature insect develops (see Fig. 2f). Furthermore, insect galls are relatively large structures (in cm scale) and usually closed (internal chamber has no opening to the outside), unlike other galls induced by animals. Insect galls were collected and taken to laboratory where they were dissected to obtain the immature and adults gall-inducing insects. It was not our objective to list the species (or taxa) of the gall-inducing insects responsible for the galls observed in the plants of our survey. For details about galling insect taxa associated with each host plant species the original studies should be consulted.

## Results

We recorded a total of 181 species of 47 families of plants that host insect galls in the state of Goiás (Table 2). Seventy-three (40.3%) plant species are registered as gall hosts for the first time to Goiás. Associated with plant species were 365 gall morphotypes, of which 192 (52.6%) had previously been recorded and 173 (47.4%) are new records. The plant families with the highest gall richness were Fabaceae, Malpighiaceae, Myrtaceae, Vochysiaceae, Sapindaceae, Erythroxylaceae, Burseraceae, Sapotaceae, Styracaceae and Asteraceae. These 10 families exhibited 58% of the insect gall morphotype richness and 52.4% of the total number of host plant species. The family Fabaceae hosted 58 gall morphotypes, while Malpighiaceae, Myrtaceae and Vochysiaceae had 29, 28 and 23 gall morphotypes, respectively.

The genera *Byrsonima* (Malpighiaceae), *Qualea* (Vochysiaceae) and *Myrcia* (Myrtaceae) were the richest in gall morphotypes, with 22, 21 and 17 morphotypes, respectively. *Byrsonima pachyphylla* A. Juss. (Malpighiaceae), *Protium heptaphyllum* March. (Burseraceae), *Qualea parviflora* Mart. (Vochysiaceae), and *Styrax pohlii* A.DC. (Styracaceae) were the host species with the most diverse gall morphotypes. Other important host species were: *Caryocar brasiliense* Cambess. (Caryocaraceae) (7), *Qualea multiflora* Mart. (Vochysiaceae) (7), *Roupala montana* Aubl. (Proteaceae) (7), *Qualea grandiflora* Mart. (Vochysiaceae) (6) and *Siparuna guianensis* Aubl. (Siparunaceae) (6).

## Discussion

We have systematically compiled the results of studies on plant species that host insect galls in the Cerrado of the state of Goiás for the first time, which resulted in new records of 73 (40.3%) plant species and 173 (47.4%) gall morphospecies. Our results corroborate previous studies that indicate Fabaceae as the plant family that hosts the greatest diversity of galling insects in the Neotropical Region (Gagné 1994, Maia and Fernandes 2004, Araújo et al. 2014b, Fernandes and Santos 2014). Local studies in different regions of Brazil found the same pattern (Gonçalves-Alvim and Fernandes 2001, Santos et al. 2010, Araújo et al. 2011, Araújo et al. 2013, Araújo et al. 2014a). Fabaceae is the most diverse plant family of the Cerrado with nearly 800 species, and so it is not surprising that it hosts the highest diversity of insect galls (Araújo et al. 2014b).

We found that *Byrsonima*, *Qualea* and *Myrcia* were the genera hosting the greatest number of gall morphotypes. These results differ from that observed in other regions of Brazil. For example, Mendonça 2007] found the genera *Mikania* (Asteraceae), *Eugenia* (Myrtaceae) and *Guapira* (Nyctaginaceae) to host the greatest diversity of galls in the state of Rio Grande do Sul in Southern Brazil. In the Brazilian Southeast, the genera *Myrcia* (Myrtaceae), *Ocotea* (Lauraceae) and *Paullinia* (Sapindaceae) hosted the most diversity of galls in the state of São Paulo (Maia et al. 2008), while *Baccharis* (Asteraceae) hosted the greatest richness of galls in state of Minas Gerais (Fernandes et al. 1996). With regards to host plant species, some species stand out as super-hosts of insect galls in Goiás, such as *Byrsonima pachyphylla*, *Protium heptaphyllum*, *Qualea parviflora* and *Styrax pohlii*. In a previous study, Maia and Fernandes 2004] recorded seven morphotypes of galls on *P. heptaphyllum* in Cerrado areas of Minas Gerais. Our compilation also adds new gall records for some host species. Galls on *Siparuna guianensis* were registered for the first time in the Neotropics by Santos et al. 2010] with only one gall morphotype, while in the present compilation six gall morphotypes are associated with this host plant.

Most of the studies performed in Brazil have shown a high diversity of galling insects in the Cerrado (Gonçalves-Alvim and Fernandes 2001, Maia and Fernandes 2004, Santos et al. 2010, Araújo et al. 2011), one of the most fragmented biomes of the world (Klink and Machado 2005). To maintain this high gall diversity it is essential that the remaining fragments of Cerrado are preserved and retain a high diversity of native plants (Araújo et al. 2014b). In the state of Goiás, areas of native vegetation are very small and the majority is widely fragmented (Cunha et al. 2007). Given the eminent risk of destruction of the remaining vegetation, inventories of host plants and their associated insect galls are urgently needed to provide a foundation for further understanding these interactions. We hope that this compilation can serve as an important tool for gall inventories and provide a theoretical reference for new studies in the state of Goiás and in all of Brazil.

## Figures and Tables

**Table 1.** Description of the sites and vegetation formations in which insect galls were sampled in the state of Goiás, Midwest, Brazil. 2005-2013.

**Code**	**Sampled site**	**Municipaly**	**Vegetation type**	**Geocoordinate**	**Altitude (m)**	**Reference**
1	Parque Estadual da Serra dos Pireneus	Pirenópolis	Savanna, Rock Savanna, Semidecidual Forest, Riparian Forest	15°49'S, 48°53'W	1,156	Araújo et al. 2011
2	Pedreira da Prefeitura	Pirenópolis	Savanna	15°50'S, 48°55'W	840	Unpublished
3	Reserva da UEG	Anápolis	Savanna	16°22'S, 48°56'W	1,097	Unpublished
4	Floresta Nacional de Silvânia	Silvânia	Grassland, Savanna, Semidecidual Forest, Riparian Forest	16°38'S, 48°39'W	963	Unpublished
5	Fazenda do Geraldo	Silvânia	Savanna	16°40'S, 48°18'W	837	Unpublished
6	Condomínio Itanhangá	Goiânia	Savanna	16°33'S, 49°17'W	762	Araújo et al. 2013
7	Bosque Saint Hilaire	Goiânia	Semidecidual Forest	16°36'S, 49°15'W	795	Santos et al. 2010
8	Zona Rural de Senador Canedo	Senador Canedo	Savanna	16°43'S, 49°06'W	774	Unpublished
9	Fazenda Bom Sucesso	Senador Canedo	Savanna	16°42'S, 49°02'W	749	Unpublished
10	Banana Menina	Hidrolânia	Savanna	16°59'S, 49°14'W	893	Unpublished
11	Zona Rural de Bela Vista	Bela Vista	Savanna	15°57'S, 48°56'W	809	Unpublished
12	Condomínio Del Rey	Caldas Novas	Savanna	17°42'S, 48°38'W	702	Santos et al. 2012
13	Fazenda Caiapônia	Caiapônia	Semidecidual Forest	16°56'S, 51°49'W	703	Unpublished
14	Fazenda Lajeado	Jataí	Savanna	17°53'S, 51°38'W	756	Unpublished
15	Estação Ecológica da UFG	Jataí	Semidecidual Forest	17°56'S, 51°42'W	646	Unpublished
16	Jardim Botânico Mata do Açude	Jataí	Semidecidual Forest	17°56'S, 51°43'W	618	Unpublished
17	Parque Nacional das Emas	Mineiros	Savanna, Grassland	17°56'S, 52°56'W	878	Araújo et al. 2014a
18	REBIO Contagem	Brasília	Savanna	15°37'S, 47°52'W	994	Unpublished
19	APA Cafuringa	Brasília	Savanna	15°31'S, 47°57'W	873	Unpublished

**Table 2.** Host plants (families and species) and insect galls (occurrence organ, form and color) recorded in the state of Goiás, Central-western Brazil. 2005-2013. References: A = Santos et al. 2010; B = Araújo et al. 2011; C = Santos et al. 2012; D = Araújo et al. 2014a; * = new record.

**Host family**	**Host species**	**Plant organ**	**Gall form**	**Gall color**	**Reference**
Anacardiaceae	*Anacardium humile* A.St.-Hil.	Leaf	Conical	Green	B
Anacardiaceae	*Anacardium occidentale* L.	Leaf	Discoid	Green	*
Anacardiaceae	*Anacardium occidentale* L.	Leaf	Globose	Brown	*
Anacardiaceae	*Astronium graveolens* Jacq.	Leaf	Globose	Red	*
Anacardiaceae	*Astronium graveolens* Jacq.	Stem/Leaf	Globose	Yellow	*
Anacardiaceae	*Litharea molleoides* (Vell.) Engl.	Leaf	Discoid	Green	*
Annonaceae	*Annona coriacea* Mart.	Leaf	Discoid	Yellow	B; D
Annonaceae	*Annona coriacea* Mart.	Leaf	Globose	Green	B; D
Annonaceae	*Annona coriacea* Mart.	Midvein	Ellipsoid	Brown	B; D
Annonaceae	*Bocageopsis mattogrossensis* (R.E.Fr.) R.E.Fr.	Leaf	Discoid	Yellow	D
Annonaceae	*Duguetia furfuracea* (A. St.-Hil.) Saff.	Leaf	Globose	Green	*
Annonaceae	*Xylopia aromatica* (Lam.) Mart.	Leaf	Discoid	Brown	*
Apocynaceae	*Aspidosperma macrocarpon* Mart.	Leaf	Discoid	Yellow	D
Apocynaceae	*Aspidosperma macrocarpon* Mart.	Leaf	Conical	Green	D
Apocynaceae	*Aspidosperma nobile* Müll.Arg.	Leaf	Discoid	Green	D
Apocynaceae	*Aspidosperma tomentosum* Mart.	Leaf	Discoid	Yellow	B
Araliaceae	*Schefflera macrocarpa* (Seem) D.C. Frodin	Leaf	Discoid	Bege	*
Araliaceae	*Schefflera macrocarpa* (Seem) D.C. Frodin	Leaf	Globose	Yellow	*
Araliaceae	*Schefflera morototoni* Aubl.	Leaf	Ellipsoid	Green	A
Araliaceae	*Schefflera vinosa* (Cham. & Schltdl.) Frodin & Fiaschi	Midvein	Ellipsoid	Brown	D
Araliaceae	*Schefflera vinosa* (Cham. & Schltdl.) Frodin & Fiaschi	Petiole	Ellipsoid	Brown	D
Asteraceae	*Eremanthus erythropappus* (DC.) MacLeish	Leaf	Globoid	Green	D
Asteraceae	*Eremanthus goyazensis* (Gardner) Sch. Bip.	Stem	Ellipsoid	Rust	*
Asteraceae	*Eremanthus* sp.	Midvein	Globose	Brown	*
Asteraceae	*Heterocondylus alatus* (Vell.) King and H. Rob.	Leaf	Ellipsoid	Green	*
Asteraceae	*Piptocarpha rotundifolia* (Less.) Baker	Leaf	Discoid	Green	D
Asteraceae	*Piptocarpha rotundifolia* (Less.) Baker	Leaf	Globose	Bege	*
Asteraceae	*Piptocarpha rotundifolia* (Less.) Baker	Midvein	Ellipsoid	Green	D
Asteraceae	*Piptocarpha rotundifolia* (Less.) Baker	Stem	Globose	Brown	*
Asteraceae	*Vernonia polysphaera* Baker	Leaf	Discoid	Yellow	*
Bignoniaceae	*Arrabidaea* sp.	Leaf/Stem	Globose	Yellow	A
Bignoniaceae	*Arrabidaea* sp.	Stem	Ellipsoid	Green	A; B
Bignoniaceae	*Handroanthus ochraceus* (Cham.) Mattos	Leaf	Conical	Brown	D
Bignoniaceae	*Handroanthus ochraceus* (Cham.) Mattos	Stem	Globose	Green	*
Bignoniaceae	*Macfadyena* sp.	Stem	Ellipsoid	Green	*
Bignoniaceae	*Tabebuia aurea* (Manso) Benth. and Hook. f. ex S. Moore	Leaf	Ellipsoid	Green	*
Bignoniaceae	*Tabebuia aurea* (Manso) Benth. and Hook. f. ex S. Moore	Leaf	Globose	Yellow	*
Bignoniaceae	*Tabebuia* sp.	Leaf	Conical	Green	C
Boraginaceae	*Cordia sellowiana* Cham.	Leaf	Globose	Brown	*
Burseraceae	*Protium heptaphyllum* March.	Leaf	Marginal roll	Green	*
Burseraceae	*Protium heptaphyllum* March.	Leaf	Cylindrical	Green	*
Burseraceae	*Protium heptaphyllum* March.	Leaf	Conical	Green	B
Burseraceae	*Protium heptaphyllum* March.	Leaf	Discoid	Green	*
Burseraceae	*Protium heptaphyllum* March.	Leaf	Globose	Brown	*
Burseraceae	*Protium heptaphyllum* March.	Leaf	Globose	Green	A
Burseraceae	*Protium heptaphyllum* March.	Midvein	Ellipsoid	Brown	A
Burseraceae	*Protium heptaphyllum* March.	Stem	Globose	Green	*
Burseraceae	*Protium* sp.	Leaf	Ellipsoid	Green	*
Burseraceae	*Protium* sp.	Leaf/Stem	Ellipsoid	Green	*
Burseraceae	*Protium* sp.	Stem	Ellipsoid	Green	*
Calophyllaceae	*Kielmeyera coriacea* Mart. & Zucc.	Leaf	Globoid	Brown	D
Calophyllaceae	*Kielmeyera coriacea* Mart. & Zucc.	Leaf	Ellipsoid	Brown	*
Calophyllaceae	*Kielmeyera coriacea* Mart. & Zucc.	Midvein	Ellipsoid	Brown	D
Calophyllaceae	*Kielmeyera coriacea* Mart. & Zucc.	Midvein	Amorphous	Brown	D
Calophyllaceae	*Kielmeyera grandiflora* (Wawra) Saddi	Leaf	Discoid	Brown	D
Calophyllaceae	*Kielmeyera rubriflora* Camb.	Leaf	Discoid	Brown	*
Calophyllaceae	*Kielmeyera* sp.	Leaf	Globose	Green	*
Caryocaraceae	*Caryocar brasiliense* Cambess.	Leaf	Discoid	Yellow	C
Caryocaraceae	*Caryocar brasiliense* Cambess.	Leaf	Discoid	Brown	*
Caryocaraceae	*Caryocar brasiliense* Cambess.	Leaf	Ellipsoid	Yellow	*
Caryocaraceae	*Caryocar brasiliense* Cambess.	Leaf	Globose	Yellow	B; D
Caryocaraceae	*Caryocar brasiliense* Cambess.	Leaf	Globoid	Yellow	B; C; D
Caryocaraceae	*Caryocar brasiliense* Cambess.	Petiole	Globoid	Brown	*
Caryocaraceae	*Caryocar brasiliense* Cambess.	Stem	Amorphous	Brown	C
Caryocaraceae	*Caryocar glabrum* (Aubl.) Pers.	Leaf	Globose	Green	*
Celastraceae	*Cheiloclinium cognatum* (Miers) A.C. Sm.	Midvein	Globose	Brown	*
Celastraceae	*Maytenus* sp.	Leaf	Discoid	Green	*
Celastraceae	*Maytenus* sp.	Stem	Ellipsoid	Brown	*
Celastraceae	*Plenckia populnea* Reissek	Leaf	Discoid	Brown	*
Chrysobalanaceae	*Couepia grandiflora* (Mart. and Zucc.) Benth. and Hook.	Leaf	Discoid	Yellow	*
Chrysobalanaceae	*Hirtella glandulosa* Spreng.	Leaf	Globose	Yellow	*
Chrysobalanaceae	*Hirtella* sp.	Leaf	Globose	Brown	*
Chrysobalanaceae	*Licania humilis* Cham. & Schltdl.	Leaf	Marginal roll	Green	*
Chrysobalanaceae	*Licania humilis* Cham. & Schltdl.	Leaf	Globose	Brown	*
Chrysobalanaceae	*Licania humilis* Cham. & Schltdl.	Midvein	Globoid	Brown	D
Chrysobalanaceae	*Licania tomentosa* Benth.	Leaf	Discoid	Green	A
Clusiaceae	*Calophylum brasiliensis* Camb.	Leaf	Globose	Green	B
Clusiaceae	*Clusia* sp.	Leaf	Amorphous	Red	B
Combretaceae	*Terminalia argentea* Mart. and Zucc.	Leaf	Discoid	Green	C
Combretaceae	*Terminalia argentea* Mart. and Zucc.	Leaf	Globose	Brown	B
Connaraceae	*Connarus suberosus* Planch.	Leaf	Discoid	Green	D
Connaraceae	*Connarus suberosus* Planch.	Leaf	Globose	Brown	C
Connaraceae	*Connarus suberosus* Planch.	Midvein	Ellipsoid	Brown	D
Connaraceae	*Connarus suberosus* Planch.	Stem	Globose	Brown	*
Connaraceae	*Rourea induta* Planch.	Leaf	Discoid	Green	D
Connaraceae	*Rourea induta* Planch.	Leaf	Discoid	Red	*
Dichapetalaceae	*Tapura* sp.	Leaf	Globose	Green	*
Dilleniaceae	*Davilla elliptica* A.St.-Hil.	Leaf	Discoid	Brown	B; C; D
Dilleniaceae	*Davilla elliptica* A.St.-Hil.	Leaf	Ellipsoid	Green	B; C
Dilleniaceae	*Davilla elliptica* A.St.-Hil.	Leaf	Discoid	Green	D
Ebenaceae	*Diospyros burchelii* Hern.	Leaf	Amorphous	Green	B
Ebenaceae	*Diospyros hispida* A.DC.	Leaf	Globose	Yellow	C
Ebenaceae	*Diospyros hispida* A.DC.	Leaf	Discoid	Yellow	D
Ebenaceae	*Diospyros hispida* A.DC.	Leaf	Globoid	Yellow	D
Ebenaceae	*Diospyros hispida* A.DC.	Stem	Globose	Green	C
Erythroxylaceae	*Erythroxylum campestre* A. St.-Hil.	Leaf	Discoid	Green	*
Erythroxylaceae	*Erythroxylum deciduum* A. St.-Hil.	Leaf	Discoid	Green	*
Erythroxylaceae	*Erythroxylum deciduum* A. St.-Hil.	Terminal bud	Globose	Brown	C
Erythroxylaceae	*Erythroxylum engleri* O.E.Schulz	Leaf	Discoid	Yellow	D
Erythroxylaceae	*Erythroxylum* sp.	Leaf	Ellipsoid	Yellow	*
Erythroxylaceae	*Erythroxylum* sp.	Stem	Globose	Brown	*
Erythroxylaceae	*Erythroxylum suberosum* A.St.-Hil.	Leaf	Amorphous	Red	B; C
Erythroxylaceae	*Erythroxylum suberosum* A.St.-Hil.	Leaf	Discoid	Green	*
Erythroxylaceae	*Erythroxylum suberosum* A.St.-Hil.	Leaf	Marginal roll	Green	C
Erythroxylaceae	*Erythroxylum suberosum* A.St.-Hil.	Leaf	Globose	Red	*
Erythroxylaceae	*Erythroxylum suberosum* A.St.-Hil.	Leaf	Marginal leaf roll	Green	D
Erythroxylaceae	*Erythroxylum tortuosum* Mart.	Leaf	Discoid	Brown	*
Erythroxylaceae	*Erythroxylum tortuosum* Mart.	Leaf	Globose	Red	*
Euphorbiaceae	*Manihot* sp.	Leaf	Conical	Red	A
Euphorbiaceae	*Manihot tripartita* (Spreng.) Müll. Arg.	Leaf	Conical	Green	C
Euphorbiaceae	*Maprounea guianensis* Aubl.	Stem	Ellipsoid	Green	*
Fabaceae	*Acosmium dasycarpum* (Vogel) Yakovlev	Leaf	Discoid	Yellow	C
Fabaceae	*Acosmium dasycarpum* (Vogel) Yakovlev	Leaf	Discoid	Green	B
Fabaceae	*Anadenanthera falcata* (Benth.) Speg.	Leaf	Globoid	Red	D
Fabaceae	*Anadenanthera peregrina* (L.) Spreng.	Leaf	Conical	Yellow	*
Fabaceae	*Anadenanthera peregrina* (L.) Spreng.	Leaf	Globose	Red	B
Fabaceae	*Andira cujabensis* Benth.	Leaf	Discoid	Green	D
Fabaceae	*Andira cujabensis* Benth.	Leaf	Conical	Green	D
Fabaceae	*Andira cujabensis* Benth.	Midvein	Ellipsoid	Brown	D
Fabaceae	*Andira cujabensis* Benth.	Petiole	Globoid	Brown	D
Fabaceae	*Andira paniculata* Benth.	Leaf	Amorphous	Green	B
Fabaceae	*Andira paniculata* Benth.	Leaf	Discoid	Brown	B
Fabaceae	*Andira paniculata* Benth.	Leaf	Ellipsoid	Green	B
Fabaceae	*Andira* sp.	Leaf	Discoid	Green	*
Fabaceae	*Andira* sp.	Leaf	Ellipsoid	Green	*
Fabaceae	*Andira* sp.	Leaf	Globose	Green	C
Fabaceae	*Bauhinia curvula* Benth.	Leaf	Globose	Yellow	*
Fabaceae	*Bauhinia curvula* Benth.	Leaf	Globose	Brown	*
Fabaceae	*Bauhinia curvula* Benth.	Leaf	Globose	Green	*
Fabaceae	*Bauhinia curvula* Benth.	Stem	Ellipsoid	Brown	*
Fabaceae	*Bauhinia* sp.1	Leaf	Globose	red	*
Fabaceae	*Bauhinia* sp.1	Stem	Ellipsoid	Brown	A
Fabaceae	*Bauhinia* sp.1	Stem	Globose	Brown	*
Fabaceae	*Bauhinia* sp.2	Leaf	Globose	Brown	C
Fabaceae	*Bauhinia* sp.3	Leaf	Globose	Branca	*
Fabaceae	*Bauhinia* sp.3	Leaf	Globose	Green	C
Fabaceae	*Bauhinia* sp.4	Leaf	Globose	Green	*
Fabaceae	*Bauhinia ungulata* L.	Leaf	Discoid	Green	A
Fabaceae	*Bauhinia ungulata* L.	Midvein	Globose	Red	B
Fabaceae	*Bowdichia virgilioides* Kunth	Leaf	Discoid	Green	D
Fabaceae	*Bowdichia virgilioides* Kunth	Midvein	Ellipsoid	Green	D
Fabaceae	*Copaifera langsdorffii* Desf.	Leaf	Bilobed	Brown	C
Fabaceae	*Copaifera langsdorffii* Desf.	Leaf	Discoid	Yellow	C
Fabaceae	*Copaifera langsdorffii* Desf.	Leaf	Globose	Yellow	*
Fabaceae	*Copaifera langsdorffii* Desf.	Leaf	Globose	Brown	*
Fabaceae	*Copaifera langsdorffii* Desf.	Stem	Ellipsoid	Brown	*
Fabaceae	*Dalbergia miscolobium* Benth.	Leaf	Discoid	Yellow	*
Fabaceae	*Dimorphandra mollis* Benth.	Leaf	Discoid	Yellow	*
Fabaceae	*Hymenaea stigonocarpa* Mart. ex Hayne	Leaf	Discoid	Brown	B
Fabaceae	*Hymenaea stigonocarpa* Mart. ex Hayne	Leaf	Discoid	Yellow	A; D
Fabaceae	*Inga cylindrica* (Vell.) Mart.	Leaf	Cylindrical	Green	A
Fabaceae	*Inga cylindrica* (Vell.) Mart.	Leaf	Globose	Green	A
Fabaceae	*Inga cylindrica* (Vell.) Mart.	Midvein	Globose	Green	A
Fabaceae	*Inga marginata* Willd.	Leaf	Discoid	Green	*
Fabaceae	*Inga* sp.	Leaf	Globose	Brown	*
Fabaceae	*Inga* sp.	Stem	Ellipsoid	Brown	*
Fabaceae	*Inga uruguensis* Hooker et Arnott	Leaf	Globose	Green	A
Fabaceae	*Leotolobium dasycarpum* Vogel	Leaf	Discoid	Green	D
Fabaceae	*Leotolobium dasycarpum* Vogel	Midvein	Ellipsoid	Green	D
Fabaceae	*Machaerium opacum* Vog.	Leaf	Discoid	Brown	*
Fabaceae	*Machaerium opacum* Vog.	Leaf	Discoid	Green	*
Fabaceae	*Piptadenia* sp.	Leaf	Discoid	Yellow	A
Fabaceae	*Piptadenia* sp.	Stem	Ellipsoid	Grey	*
Fabaceae	*Sclerolobium paniculatum* Vog.	Leaf	Discoid	Green	*
Fabaceae	*Stryphnodendron adstringens* (Mart.) Coville	Leaf	Discoid	Brown	*
Fabaceae	*Stryphnodendron adstringens* (Mart.) Coville	Leaf	Discoid	Yellow	D
Fabaceae	*Stryphnodendron adstringens* (Mart.) Coville	Midvein	Ellipsoid	Brown	D
Fabaceae	*Tachigali aurea* Tul.	Leaf	Ellipsoid	Brown	D
Fabaceae	*Tachigali vulgaris* L.G.Silva & H.C.Lima	Leaf	Globoid	Yellow	D
Lauraceae	*Nectandra cuspidata* Nees	Leaf	Discoid	Green	A
Loganiaceae	*Strychnos pseudoquina* A. St.-Hil.	Leaf	Discoid	Green	*
Loganiaceae	*Strychnos pseudoquina* A. St.-Hil.	Leaf	Globose	Green	C
Loranthaceae	*Struthanthus* sp.	Leaf	Discoid	Brown	B
Malpighiaceae	*Banisteriopsis argyrophylla* (A. Juss.) B. Gates	Leaf	Globose	Green	*
Malpighiaceae	*Banisteriopsis megaphylla* (A. Juss.) B. Gates	Leaf	Globose	Green	*
Malpighiaceae	*Byrsonima basiloba* A. Juss.	Leaf	Globose	Brown	*
Malpighiaceae	*Byrsonima coccolobifolia* Kunth	Leaf	Conical	Green	D
Malpighiaceae	*Byrsonima coccolobifolia* Kunth	Leaf	Discoid	Yellow	D
Malpighiaceae	*Byrsonima coccolobifolia* Kunth	Leaf	Discoid	Brown	D
Malpighiaceae	*Byrsonima guilleminiana* Brad. and Mark.	Leaf	Discoid	Yellow	B
Malpighiaceae	*Byrsonima laxiflora* Griseb.	Leaf	Conical	Yellow	*
Malpighiaceae	*Byrsonima laxiflora* Griseb.	Stem	Ellipsoid	Brown	*
Malpighiaceae	*Byrsonima pachyphylla* A. Juss.	Leaf	Conical	Yellow	B
Malpighiaceae	*Byrsonima pachyphylla* A. Juss.	Leaf	Discoid	Brown	*
Malpighiaceae	*Byrsonima pachyphylla* A. Juss.	Leaf	Ellipsoid	Brown	*
Malpighiaceae	*Byrsonima pachyphylla* A. Juss.	Leaf	Vírgula	Yellow	*
Malpighiaceae	*Byrsonima pachyphylla* A. Juss.	Leaf	Conical	Brown	D
Malpighiaceae	*Byrsonima pachyphylla* A. Juss.	Leaf	Discoid	Green	D
Malpighiaceae	*Byrsonima pachyphylla* A. Juss.	Stem	Ellipsoid	Brown	*
Malpighiaceae	*Byrsonima pachyphylla* A. Juss.	Stem	Globose	Brown	C
Malpighiaceae	*Byrsonima* sp.1	Leaf	Conical	Green	*
Malpighiaceae	*Byrsonima* sp.2	Leaf	Discoid	Green	*
Malpighiaceae	*Byrsonima* sp.3	Leaf	Conical	Yellow	B
Malpighiaceae	*Byrsonima* sp.3	Midvein	Ellipsoid	Green	*
Malpighiaceae	*Byrsonima verbascifolia* (L.) DC.	Leaf	Discoid	Green	D
Malpighiaceae	*Byrsonima verbascifolia* (L.) DC.	Leaf	Conical	Yellow	*
Malpighiaceae	*Byrsonima verbascifolia* (L.) DC.	Leaf	Globose	Purple	*
Malpighiaceae	*Diplopterys pubipetala* (A. Juss.) W.R. Anderson and C. Cav. Davis	Leaf	Discoid	Green	*
Malpighiaceae	*Heteropterys byrsonimifolia* A. Juss.	Leaf	Discoid	Brown	*
Malpighiaceae	*Heteropterys byrsonimifolia* A. Juss.	Stem	Ellipsoid	Brown	*
Malpighiaceae	*Peixotoa* sp.	Leaf	Globose	Green	C
Malpighiaceae	*Pterandra pyroidea* A. Juss.	Leaf	Globose	Yellow	B
Malvaceae	*Eriotheca gracilipes* (K.Schum.) A.Robyns	Leaf	Discoid	Yellow	*
Malvaceae	*Eriotheca gracilipes* (K.Schum.) A.Robyns	Leaf	Discoid	Green	D
Malvaceae	*Eriotheca gracilipes* (K.Schum.) A.Robyns	Midvein	Ellipsoid	Green	D
Malvaceae	*Eriotheca gracilipes* (K.Schum.) A.Robyns	Midvein	Globoid	Brown	D
Malvaceae	*Eriotheca pubescens* (Mart. & Zucc.) Schott & Endl.	Leaf	Globoid	Brown	C
Malvaceae	*Eriotheca pubescens* (Mart. & Zucc.) Schott & Endl.	Leaf	Discoid	Brown	*
Malvaceae	*Eriotheca pubescens* (Mart. & Zucc.) Schott & Endl.	Leaf	Discoid	Green	*
Malvaceae	*Pseudobombax longiflorum* (Mart. and Zucc.) A. Robyns	Leaf	Conical	Red	B; C
Malvaceae	*Sida micrantha* A.St.-Hil	Leaf/Stem	Globose	Yellow	B
Melastomataceae	*Macairea radula* (Bonpl.) DC.	Leaf/Stem	Globose	Yellow	B
Melastomataceae	*Miconia albicans* (Sw.) Triana	Leaf	Globoid	Brown	B; C; D
Melastomataceae	*Miconia albicans* (Sw.) Triana	Leaf	Discoid	Brown	D
Melastomataceae	*Miconia albicans* (Sw.) Triana	Leaf	Discoid	Bege	*
Melastomataceae	*Miconia albicans* (Sw.) Triana	Leaf	Globose	Brown	*
Melastomataceae	*Miconia* sp.1	Stem	Globose	Green	*
Melastomataceae	*Miconia* sp.2	Leaf	Amorphous	Brown	*
Melastomataceae	*Miconia* sp.2	Stem	Ellipsoid	Brown	*
Melastomataceae	*Miconia* sp.3	Inflorescence	Globose	Brown	*
Meliaceae	*Guarea* sp.	Stem	Globose	Brown	*
Myristicaceae	*Virola sebifera* Aubl.	Leaf	Globose	Green	*
Myrsinaceae	*Rapanea guianensis* Aubl.	Stem	Ellipsoid	Brown	*
Myrtaceae	*Campomanesia adamantium* (Cambess.) O.Berg	Leaf	Discoid	Brown	D
Myrtaceae	*Eugenia aurata* O.Berg	Leaf	Discoid	Black	D
Myrtaceae	*Eugenia punicifolia* (Kunth) DC.	Leaf	Ellipsoid	Green	B
Myrtaceae	*Eugenia* sp.	Leaf	Discoid	Green	*
Myrtaceae	*Eugenia ternatifolia* Cambess.	Leaf	Globoid	Green	D
Myrtaceae	*Myrcia bella* Cambess.	Leaf	Discoid	Brown	D
Myrtaceae	*Myrcia bella* Cambess.	Midvein	Ellipsoid	Green	D
Myrtaceae	*Myrcia camapuanensis* N.Silveira	Midvein	Globoid	Brown	D
Myrtaceae	*Myrcia guianensis* (Aubl.) DC.	Leaf	Globoid	Green	D
Myrtaceae	*Myrcia guianensis* (Aubl.) DC.	Leaf	Discoid	Green	D
Myrtaceae	*Myrcia guianensis* (Aubl.) DC.	Midvein	Globoid	Brown	D
Myrtaceae	*Myrcia guianensis* (Aubl.) DC.	Midvein	Conical	Green	D
Myrtaceae	*Myrcia multiflora* (Lam.) DC.	Leaf	Globoid	Green	D
Myrtaceae	*Myrcia rostrata* DC	Leaf	Discoid	Green	A
Myrtaceae	*Myrcia* sp.1	Leaf	Globose	Green	B
Myrtaceae	*Myrcia* sp.1	Stem	Ellipsoid	Brown	*
Myrtaceae	*Myrcia* sp.1	Stem	Globose	Green	*
Myrtaceae	*Myrcia* sp.2	Leaf	Globose	Green	A
Myrtaceae	*Myrcia* sp.2	Stem	Ellipsoid	Brown	*
Myrtaceae	*Myrcia* sp.3	Axillary bud	Ellipsoid	Brown	*
Myrtaceae	*Myrcia variabilis* DC.	Leaf	Discoid	Brown	D
Myrtaceae	*Myrcia vestita* DC.	Leaf	Discoid	Yellow	D
Myrtaceae	*Psidium laruotteanum* Cambess.	Leaf	Globoid	Brown	D
Myrtaceae	*Psidium laruotteanum* Cambess.	Leaf	Discoid	Brown	D
Myrtaceae	*Psidium laruotteanum* Cambess.	Leaf	Marginal leaf roll	Green	D
Myrtaceae	*Psidium myrtoides* O.Berg	Leaf	Discoid	Yellow	*
Myrtaceae	*Psidium myrtoides* O.Berg	Stem	Ellipsoid	Brown	*
Myrtaceae	*Psidium salutare var. pohlianum* (O.Berg) Landrum	Leaf	Globose	Green	B
Nyctaginaceae	*Guapira gracilifolia* (Vell.) Reitz	Leaf	Discoid	Brown	*
Nyctaginaceae	*Guapira noxia* (Netto) Lundell	Leaf	Discoid	Brown	D
Nyctaginaceae	*Guapira noxia* (Netto) Lundell	Midvein	Ellipsoid	Brown	D
Nyctaginaceae	*Guapira noxia* (Netto) Lundell	Midvein	Globoid	Brown	D
Nyctaginaceae	*Guapira* sp.	Leaf	Discoid	Yellow	*
Nyctaginaceae	*Guapira* sp.	Leaf	Globose	Green	*
Nyctaginaceae	*Guapira* sp.	Midvein	Ellipsoid	Green	*
Nyctaginaceae	*Guapira* sp.	Stem	Ellipsoid	Brown	*
Nyctaginaceae	*Neea theifera* Oerst.	Leaf	Discoid	Green	C
Ochnaceae	*Ouratea hexasperma* (A.St.-Hil.) Baill.	Leaf	Discoid	Green	B; C; D
Ochnaceae	*Ouratea hexasperma* (A.St.-Hil.) Baill.	Stem	Globose	Brown	*
Ochnaceae	*Ouratea spectabilis* (Mart.) Engl.	Leaf	Conical	Brown	D
Ochnaceae	*Ouratea spectabilis* (Mart.) Engl.	Leaf	Discoid	Green	D
Piperaceae	*Piper arboreum* Aubl.	Leaf	Discoid	Green	A
Piperaceae	*Piper arboreum* Aubl.	Stem	Ellipsoid	marron	A
Piperaceae	*Piper arboreum* Aubl.	Stem	Globose	Green	B
Piperaceae	*Piper* sp.	Leaf	Globose	Green	*
Proteaceae	*Roupala montana* Aubl.	Leaf	Discoid	Green	B; C; D
Proteaceae	*Roupala montana* Aubl.	Leaf	Conical	Green	C
Proteaceae	*Roupala montana* Aubl.	Leaf	Ellipsoid	Brown	*
Proteaceae	*Roupala montana* Aubl.	Leaf	Ellipsoid	Green	*
Proteaceae	*Roupala montana* Aubl.	Midvein	Ellipsoid	Green	D
Proteaceae	*Roupala montana* Aubl.	Petiole	Ellipsoid	Red	D
Proteaceae	*Roupala montana* Aubl.	Stem	Ellipsoid	Brown	*
Ramnaceae	*Rhamnidium elaeocarpum* Reissek	Stem	Globose	Brown	*
Rubiaceae	*Alibertia edulis* (Rich.) A. Rich. Ex DC.	Leaf	Discoid	Yellow	*
Rubiaceae	*Alibertia* sp.1	Leaf	Discoid	Green	*
Rubiaceae	*Alibertia* sp.2	Leaf	Discoid	Orange	*
Rubiaceae	*Coussarea* sp.	Leaf	Globose	Yellow	*
Rubiaceae	*Coussarea* sp.	Stem	Globose	Green	*
Rubiaceae	*Palicourea rigida* Kunth	Leaf	Globoid	Brown	D
Rubiaceae	*Palicourea rigida* Kunth	Leaf	Amorphous	Bege	*
Rubiaceae	*Palicourea rigida* Kunth	Leaf	Globose	Yellow	B
Rubiaceae	*Palicourea* sp.	Stem	Ellipsoid	Brown	*
Salicaceae	*Casearia sylvestris* Sw.	Leaf	Discoid	Brown	D
Salicaceae	*Casearia sylvestris* Sw.	Leaf	Globose	Green	*
Sapindaceae	*Cupania* sp.	Stem	Ellipsoid	Green	*
Sapindaceae	*Matayba guianensis* Aubl.	Leaf	Globose	Green	*
Sapindaceae	*Matayba* sp.	Leaf	Conical	Green	*
Sapindaceae	*Matayba* sp.	Midvein	Globose	Green	*
Sapindaceae	*Serjania lethalis* A. St.-Hil.	Leaf	Discoid	Green	*
Sapindaceae	*Serjania obtusidentata* Radlk	Leaf	Discoid	Green	A
Sapindaceae	*Serjania obtusidentata* Radlk	Leaf/Stem	Ellipsoid	Green	A
Sapindaceae	*Serjania* sp.	Leaf	Discoid	Green	*
Sapindaceae	*Serjania* sp.	Leaf	Ellipsoid	Green	*
Sapindaceae	*Serjania* sp.	Leaf	Globose	Brown	*
Sapindaceae	*Serjania* sp.	Midvein	Ellipsoid	Brown	B
Sapindaceae	*Serjania* sp.	Midvein	Ellipsoid	Green	*
Sapindaceae	*Serjania* sp.	Stem	Conical	Brown	*
Sapindaceae	*Serjania* sp.	Stem	Ellipsoid	Brown	*
Sapotaceae	*Chrysophylum marginatum* (Hook. and Arn.) Radlk.	Leaf	Globose	Green	*
Sapotaceae	*Micropholis* sp.	Leaf	Globose	Green	B
Sapotaceae	*Micropholis* sp.	Stem	Globose	Brown	B
Sapotaceae	*Pouteria ramiflora* (Mart.) Radlk.	Leaf	Discoid	Brown	D
Sapotaceae	*Pouteria ramiflora* (Mart.) Radlk.	Leaf	Globose	Green	*
Sapotaceae	*Pouteria ramiflora* (Mart.) Radlk.	Stem	Ellipsoid	Brown	B
Sapotaceae	*Pouteria* sp.	Leaf	Discoid	Red	*
Sapotaceae	*Pouteria torta* (Mart.) Radlk.	Leaf	Conical	Green	D
Sapotaceae	*Pouteria torta* (Mart.) Radlk.	Leaf	Discoid	Yellow	D
Sapotaceae	*Pouteria torta* (Mart.) Radlk.	Leaf	Conical	Brown	D
Sapotaceae	*Pouteria torta* (Mart.) Radlk.	Leaf	Discoid	Brown	C
Siparunaceae	*Siparuna guianensis* Aubl.	Leaf	Cylindrical	Green	*
Siparunaceae	*Siparuna guianensis* Aubl.	Leaf	Ellipsoid	Yellow	*
Siparunaceae	*Siparuna guianensis* Aubl.	Leaf	Ellipsoid	Brown	*
Siparunaceae	*Siparuna guianensis* Aubl.	Stem	Ellipsoid	Green	*
Siparunaceae	*Siparuna guianensis* Aubl.	Stem	Globose	Brown	*
Siparunaceae	*Siparuna guianensis* Aubl.	Stem	Globose	Green	A
Siparunaceae	*Siparuna* sp.	Midvein	Ellipsoid	Green	*
Smilacaceae	*Smilax fluminensis* Steud.	Leaf	Discoid	Yellow	*
Smilacaceae	*Smilax fluminensis* Steud.	Leaf	Globose	Green	*
Smilacaceae	*Smilax* sp.	Leaf	Globose	Green	B
Solanaceae	*Solanum* sp.	Stem	Ellipsoid	Brown	*
Styracaceae	*Styrax acuminatus* Pohl.	Leaf	Discoid	Brown	*
Styracaceae	*Styrax acuminatus* Pohl.	Stem	Ellipsoid	Brown	*
Styracaceae	*Styrax ferrugineus* Nees and Mart.	Leaf	Discoid	Green	*
Styracaceae	*Styrax ferrugineus* Nees and Mart.	Stem	Ellipsoid	Brown	C
Styracaceae	*Styrax pohlii* A.DC.	Leaf	Conicle	Green	*
Styracaceae	*Styrax pohlii* A.DC.	Leaf	Globoid	Brown	A
Styracaceae	*Styrax pohlii* A.DC.	Leaf	Globoid	Green	B
Styracaceae	*Styrax pohlii* A.DC.	Leaf	Erythrocyte	Brown	A
Styracaceae	*Styrax pohlii* A.DC.	Leaf	Globoid (abaxial)	Brown	A
Styracaceae	*Styrax pohlii* A.DC.	Leaf	Discoid (adaxial)	Brown	B
Styracaceae	*Styrax pohlii* A.DC.	Stem	Fusiform	Brown	A; B
Styracaceae	*Styrax pohlii* A.DC.	Stem	Globoid	Brown	A; B
Styracaceae	*Styrax* sp.	Leaf	Globose	Green	B
Ulmaceae	*Celtis iguanaea* (Jacq.) Sarg.	Leaf	Discoid	Yellow	A
Ulmaceae	*Celtis iguanaea* (Jacq.) Sarg.	Leaf/Stem	Conical	Green	*
Ulmaceae	*Trema micrantha* (L.) Blume	Leaf	Ellipsoid	White	A
Ulmaceae	*Trema micrantha* (L.) Blume	Leaf	Globose	Green	A
Ulmaceae	*Trema micrantha* (L.) Blume	Stem	Globose	Brown	A
Verbenaceae	*Aegiphyla* sp.	Leaf	Globose	Green	*
Vochysiaceae	*Qualea grandiflora* Mart.	Leaf	Discoid	Green	B; C
Vochysiaceae	*Qualea grandiflora* Mart.	Leaf	Globoid	Green	D
Vochysiaceae	*Qualea grandiflora* Mart.	Leaf	Conical	Green	D
Vochysiaceae	*Qualea grandiflora* Mart.	Leaf	Discoid	Brown	C
Vochysiaceae	*Qualea grandiflora* Mart.	Leaf	Globose	Brown	*
Vochysiaceae	*Qualea grandiflora* Mart.	Stem	Globose	Brown	C
Vochysiaceae	*Qualea multiflora* Mart.	Leaf	Discoid	Green	D
Vochysiaceae	*Qualea multiflora* Mart.	Leaf	Discoid	Brown	*
Vochysiaceae	*Qualea multiflora* Mart.	Leaf	Star	Green	C
Vochysiaceae	*Qualea multiflora* Mart.	Leaf	Globose	Brown	C
Vochysiaceae	*Qualea multiflora* Mart.	Leaf	Laminar	Green	*
Vochysiaceae	*Qualea multiflora* Mart.	Midvein	Ellipsoid	Brown	D
Vochysiaceae	*Qualea multiflora* Mart.	Stem	Globose	Brown	*
Vochysiaceae	*Qualea parviflora* Mart.	Leaf	Conical	Green	C; D
Vochysiaceae	*Qualea parviflora* Mart.	Leaf	Star	Green	D
Vochysiaceae	*Qualea parviflora* Mart.	Leaf	Discoid	Green	B; D
Vochysiaceae	*Qualea parviflora* Mart.	Leaf	Star	Red	B; D
Vochysiaceae	*Qualea parviflora* Mart.	Leaf	Globose	Yellow	*
Vochysiaceae	*Qualea parviflora* Mart.	Leaf	Vulcano	Green	*
Vochysiaceae	*Qualea parviflora* Mart.	Stem	Ellipsoid	Brown	*
Vochysiaceae	*Qualea parviflora* Mart.	Stem	Globose	Brown	*
Vochysiaceae	*Salvertia convallariaeodora* A.St.-Hil.	Leaf	Globose	Brown	B
Vochysiaceae	*Vochysia* sp.	Leaf	Discoid	Green	C; D

## References

[B1902542] Abrahamson Warren G., Melika George, Scrafford Robert, Csoka Gyorgy (1998). Gall-Inducing Insects Provide Insights into Plant Systematic Relationships. American Journal of Botany.

[B1902972] Araújo WS, Silva IPA, Santos BB, Gomes-Klein VL (2013). Host plants of entomogenous galls in areas of cerrado in the state of Goiás, Brazil. Acta Botanica Brasilica.

[B1902552] Araújo Walter Santos de, Santos Benedito Baptista dos, Gomes-Klein Vera Lúcia (2011). Insect galls from Serra dos Pireneus, GO, Brazil. Biota Neotropica.

[B1902562] Araújo Walter Santos de, Sobral Fernando Landa, Maracahipes Leandro (2014). Insect galls of the Parque Nacional das Emas (Mineiros, GO, Brazil). Check List.

[B1902572] Araújo Walter Santos de, Santos Benedito Baptista dos, Guilherme Frederico Augusto Guimarães, Scareli-Santos Claudia (2014). Galling Insects in the Brazilian Cerrado: Ecological Patterns and Perspectives. Neotropical Insect Galls.

[B1902586] Bregonci Juliana de Menezes, Polycarpo Polyanna Vieira, Maia Valéria Cid (2010). Galhas de insetos do Parque Estadual Paulo César Vinha (Guarapari, ES, Brasil). Biota Neotropica.

[B2183127] Carneiro Marco Antonio A., Branco Cristina S. A., Braga Carlos E. D., Almada Emmanuel D., Costa Marina B. M., Maia Valéria C., Fernandes Geraldo Wilson (2009). Are gall midge species (Diptera, Cecidomyiidae) host-plant specialists?. Revista Brasileira de Entomologia.

[B1902596] Cunha Hélida Ferreira, Ferreira AA, Brandão D (2007). Composição e fragmentação do Cerrado em Goiás usando Sistema de Informação Geográfica (SIG).. Boletim Goiano de Geografia.

[B1902995] Espírito-Santo M, Fernandes GW (2007). How many species of gall-inducing insects are there on Earth, and where are they?. Annals of Entomological Society of America.

[B1902608] Fernandes GW, Santos J (2014). Neotropical Insect Galls.

[B1902617] Fernandes G. W., Carneiro M. A.A., Lara A. C.F., Allain L. R., Andrade G. I., Julião G. R., Reis T. R., Silva I. M. (1996). Galling insects on neotropical species of Baccharis (Asteraceae). Tropical Zoology.

[B1903007] Gagné RJ (1994). The gall midges of the region neotropical. Ithaca, Comstock, USA.

[B1902631] Gonçalves-Alvim Silmary J., Fernandes Geraldo Wilson (2001). Comunidades de insetos galhadores (Insecta) em diferentes fisionomias do cerrado em Minas Gerais, Brasil. Revista Brasileira de Zoologia.

[B1902641] Klink CA, Machado RB (2005). Conservation of the Brazilian Cerrado. Conservation Biology.

[B1902651] Maia V. C., Fernandes G. W. (2004). Insect galls from Serra de São José (Tiradentes, MG, Brazil). Brazilian Journal of Biology.

[B1902661] Maia Valeria Cid, Magenta Mara Angelina Galvão, Martins Suzana Ehlin (2008). Ocorrência e caracterização de galhas de insetos em áreas de restinga de Bertioga (São Paulo, Brasil). Biota Neotropica.

[B2183140] Mani M. S. (1964). Ecology of plant galls.

[B1903027] Mendonça MS (2007). Plant diversity and galling arthropod diversity - searching for taxonomic patterns in an animal-plant interaction in the Neotropics. Boletin de la Sociedad Argentina de Botanica.

[B1902680] Moura Maria Zabelê Dantas, Soares Geraldo Luiz Gonçalves, Isaias Rosy Mary dos Santos (2008). Species-specific changes in tissue morphogenesis induced by two arthropod leaf gallers in *Lantana
camara* L. (Verbenaceae). Australian Journal of Botany.

[B2183149] Rohfritsch O., Shorthouse J. D., Rohfritsch O. (1982). Pattems in gall development. Biology of insect- and acarina-induced galls.

[B1903046] Santos BB, Ribeiro BA, Silva TM, Araújo WS (2012). Galhas de insetos em uma área de cerrado sentido restrito na região semi-urbana de Caldas Novas (Goiás, Brasil).. Revista Brasileira de Biociências.

[B1902932] Santos Benedito Baptista dos, Ferreira Heleno Dias, Araújo Walter Santos de (2010). Ocorrência e caracterização de galhas entomógenas em uma área de floresta estacional semidecídua em Goiânia, Goiás, Brasil. Acta Botanica Brasilica.

[B1902942] Stone Graham N., Schönrogge Karsten (2003). The adaptive significance of insect gall morphology. Trends in Ecology & Evolution.

